# From Justice to the Good? Liberal Utilitarianism, Climate Change and the Coronavirus Crisis

**DOI:** 10.1017/S0963180120000900

**Published:** 2020-10-09

**Authors:** HENRIK RYDENFELT

For John Rawls, it sufficed to show that people would choose his principles over utilitarian ones.^1^ After Rawls, the game appears to have changed. Utilitarianism is not in vogue in ongoing philosophical and political debates, including debates in bioethics. However, it remains influential in societal and political decisionmaking: look at the current coronavirus disease (COVID-19) pandemic that—in democratic societies at least—has prompted experts and political leaders to concentrate on attempts to maximize the health and wellbeing of citizens, often at the expense of civil rights and liberties as well as of financial profit. This makes a reconsideration of the prospects of utilitarianism most timely—in both political philosophy and bioethics. Such a reconsideration is provided by Matti Häyry^2^ in terms of both a thought-out account of his own liberal utilitarianism^3^ and a compelling overview of the main competitors in the philosophical (and, to an extent, political) debates on justice. In “Just Better Utilitarianism,” Häyry argues that, unlike competing theories of justice, liberal utilitarianism puts us in a position to deal with some matters of great urgency. In this commentary, I concentrate on the merits of this claim.

Häyry’s vision is pristine in its outline but rich in detail. His arguments for his view—and, in particular, against some long-standing objections—are complex and fresh. To keep things manageable, I concentrate on a particular line of argument Häyry presents. Liberal utilitarianism, Häyry argues, has an advantage: it is better suited to dealing with large-scale concerns threatening human and ecological wellbeing. An example is the climate crisis and its devastating consequences. Competing theories of justice, he writes, “do not spontaneously endorse downscaling on climate-related grounds. Climate action is not a primary good in Rawls’s theory, nor is it mentioned in [Martha] Nussbaum’s list of important capabilities.”^4^ I think Häyry is right in calling out many accounts of justice for their lack of consideration for some matters of urgency. It is not his only argument for the liberal utilitarian position nor the only one that could be provided. However, it appears particularly compelling: if other accounts of justice do *not* sufficiently address issues such as climate change (or, perhaps, the coronavirus crisis), and liberal utilitarianism *does*, it clearly has an advantage.

But is this so? Three questions immediately present themselves, and I will begin by considering them in turn. Are theories of justice expected to provide us with the means to deal with issues of such urgency? If they are, do not competing accounts do so? Finally, does Häyry’s liberal utilitarianism provide us with such means? After considering these questions, I conduct a bit of philosophical excavation in an attempt to unearth the different conceptions of *the good* endorsed (at least arguably) by these theories of justice. I argue that Häyry’s map of justice would benefit and be enriched by a reconsideration of this aspect. Finally, drawing from the recent example of the coronavirus crisis, in comparison to the threat of climate change, I propose that times of (perceived) crisis appear to be accompanied by an increasing concentration on the good. I add the further consideration that, while the coronavirus crisis has the makings of turning the utilitarian account all the more relevant at least for the time being, the question becomes whether, after the crisis has passed, it will remain to be as relevant in light of other global concerns such as climate change. Moreover, as I will propose, even if wellbeing does become a central concern for our societies, it is a good question whether this concern will assume a far more parochial scope than the liberal utilitarian might desire.

## Just Just Utilitarianism?

Häyry charges competing accounts of justice with a clear omission: they are not able to address large-scale issues of the good, such as the concerns brought about by climate change. But perhaps this is a feature, not a glitch. At their most basic, at least, theories of justice deal with the allocation of burdens and benefits. Perhaps they are not even intended to address concerns of the future, or to provide a perspective from which to deal with such issues; they leave issues of this kind for other political and philosophical debates. We might admit that the future good remains rather invisible in many of these accounts—but the consideration of such good is not the core business of theories of justice.

I presume that Häyry could argue that, even if this is the case, liberal utilitarianism has the added benefit of being explicit about the future good while competing accounts are not. However, if the difference is merely that other accounts of justice are too silent about future good, could they not be equipped with further refinements to deal with such large-scale issues? Unlike utilitarians such as Häyry, the defenders of Rawlsian principles or the capabilities approach have not always been quite explicit about what they consider the good to be. But a Rawls or a Nussbaum may retort that, although there is a clear controversy about what justice is based on, their views of justice can be developed in a way that puts the climate crisis on the agenda. Climate action could be included among the list of capabilities, as Häyry himself suggests. (Why not, for the list is at least open to reconstruction and interpretation in different contexts.) A Rawlsian could easily argue—and has—that climate action follows from the maximin principle.^5^ After all, it is those least well off that are disproportionately affected by the catastrophic consequences of climate change—mostly brought about by the well off. Perhaps mitigating climate change is not a section heading in *A Theory of Justice*, but neither does it stand as a principle of Häyry’s long list of the tenets of liberal utilitarianism.

I presume that, again, Häyry has an answer at hand. Even with such refinements in terms of the future good in place, the competing accounts would not address grave major concerns such as climate change *as directly.* The particular conception of the good involved in liberal utilitarianism—wellbeing—forces us to immediately consider such long-term prospects and threats. Moreover, Häyry’s need-based model is equipped to take into consideration the wellbeing of sentient beings other than humans. It seems that liberal utilitarianism would place climate action on our agenda more immediately than its competitors would. But would it?

Utilitarianism in general is, deep down, all about achieving the good; this is one thing that appears constant in its long history. It seems inherently forward-looking. J. S. Mill and Henry Sidgwick went to great lengths in attempting to show that our usual views of just recompense can be explained by utilitarian principles.^6^ Rewards give incentive for further attempts to maximize utility. These arguments may be unsuccessful. We do not seem to care about incentivizing the drive for the overall good all that much. (After all, if we did, we might be less eager to incentivize the achievement of short-term financial gain.) However, as an overall account of normative ethics, the gaze of utilitarianism is forward. I suspect that averting a great calamity such as climate catastrophe would be an ethical priority for Mill or Sidgwick.

However, is this still true in the case of utilitarianism when put to play, primarily, as an account of *justice*? This brings us back to the potential limitations of theories of justice, in general, in dealing with such problems. One issue is time. Perhaps theories of justice are inherently backward-looking. Accounts of justice fix our gaze on our current conditions and past actions. They appear locked to a presentist perspective. And how could it be otherwise? Should we be compensated for crimes of the future? (Precompensated?) Should we be rewarded for future good acts? (Prewarded?) Too complicated. Another issue concerns the recipients of justice—the question of who should get what, in terms of, mostly, benefits and burdens. Here, theories of justice consider those *who* already are and *what* they are (e.g., human). Although burdens and benefits are prospective, they are largely based on who exists and their current state, not on what will or might become (of us). It may even be that giving such an account is antithetical to their overall zeal of providing an account of justice only. Theories of justices—as they are conventionally structured and presented—easily slip out of tune with such considerations.

Indeed, inspecting again the principles of liberal utilitarianism, the connection between large-scale issues of wellbeing and justice becomes less than obvious. Häyry’s principles, as encompassing as they are, are anchored, in different ways, in the *actual* needs of *existing* human beings and other rational and sentient beings. These needs are, moreover, at least to an extent, subjectively determined by those who have them. This seems inevitable: again, as an account of justice Häyry’s liberal utilitarianism is occupied by considerations of who (and what) deserves good. The more easily the utilitarian account is able to deal with such usual questions of justice, the less it has to do with contemplating, attaining, or maximizing prospective good. It is similarly bound to at least a modicum of presentism: the anchor of justice is meeting the (basic) needs of existing people and other beings, and these needs are at least to some extent subjectively determined. Even if liberal utilitarianism bolsters a view of the good as wellbeing, it appears just as limited as its main alternatives in turning large-scale concerns to wellbeing into actionable principles of justice. Utilitarianism becomes *just* just utilitarianism.

## Theories of Justice and Accounts of the Good

My concern is that liberal utilitarianism loses its distinctiveness when turned into an account of justice. From its perspective, major threats to future good—even in terms of wellbeing—do not appear as imminently pressing as it initially might seem. Competing accounts might do just as well, at least upon further reconstruction. To be more precise, the real concern is this: *none* of the accounts of justice—liberal utilitarianism included—seems to offer *that* much by way of means to deal with large-scale issues of the good of the future.

Here is one way of making these issues more explicit. Perhaps Häyry’s picture of the theories of justice should be encircled by a conception of the good that the different accounts of justice more or less reflect. Perhaps better yet, each conception of justice could be matched by some account or accounts of the good. A further examination of the conceptions of value implicated by these accounts would be in order, even if the theories of justice are not themselves derivative of one or another account of the good.^7^ Such an examination, then, could be used to unearth the real differences between these six accounts of justice in terms of meeting such crises and challenges as climate change. The prospects of such an examination might initially appear slim. In another context, Häyry has argued that the trio of accounts of justice on the lower right side of the figure (capabilities, utilitarian, and social responsibility) are “outcome-oriented”: they are concentrated on *what* justice is to bring.^8^ Meanwhile, the other trio on the upper left side (individuals’ rights, communitarianism, and ethics of care) are more concentrated on the *who* questions of (the distribution of) burdens and benefits, and on how people are treated.

However, it may be that the map of justice does not do full justice to the way in which these latter accounts are also wedded to different conceptions of the good—even if the lower right side is more explicit about the conceptions involved. The upper-left side trio is critical of *universalized* views of the good—of anyone who pretends to know what is good for “us.” But this should not be taken to entail that they do not entail a conception of the good. They simply do not find that the good is similarly universal.

This is perhaps easiest seen on the vertical axis between capitalism and socialism, or their contemporary variations. The two differ in their conception of (the principles of) desert: one emphasizes achievement, the other need. They also differ with respect to their views on the way desert is to be put to use: one emphasizes the freedom of the individual to pursue the (largely material) goods that they deem worthwhile, while the other often entails a view of state-level actors as the best judges of what individuals really need by way of material goods. Nevertheless, they agree, at least roughly, in their conception of the good that desert consists of: material good—money, food, products, housing, and so on.

What if the analogy were continued across the map? In the lower corner on the left, Häyry has placed a number of views, including the ethics of care that is premised on the relevance of contextual responsibilities and relationships and appreciating differences, but also intersectional feminism. The calls from this corner are for further inclusion, participation, influence, and a more prominent role for different minorities in our societies. Such goods do not seem to differ in kind from the lists of capabilities advocated by those who are in the opposite corner—which Häyry labels capability promotion. The connection may appear a bit tenuous; however, this is mainly because a variety of accounts of justice in terms of relations, care, and recognition are juxtaposed with a paradigmatic list of capabilities that a limited number of thinkers have provided. (Think of the oppositions and commonalities between “second-wave” feminism, calling for universal rights to be extended to all, and intersectional feminism, critical of the idea of a universal list of rights or capabilities.) Again, the main contrast is, rather, between universal accounts of capabilities with a variety of views resisting the idea that a limited number of professional philosophers, economists, and decisionmakers would have a better idea of what is good for “us.” Nevertheless, there appears to be a common core of good at issue.

Finally, the most unexpected commonality may be between the final “opposites” in the figure, utilitarianism and the communal tradition. The contrast between the conservative, inward-looking views with an emphasis on responsibilities toward one’s community, and universal utilitarianism—especially in its Häyryan rendition—is striking. However, again, the two views seem to share their basic conception of the good as wellbeing, in the case of the communal tradition, in terms of the benefit of the community broadly speaking. Again, the difference is not in the notion of good, but between a universal (or universalizable) and a local account. The utilitarian attempts to maximize wellbeing universally, relying both on subjective opinion and the testimony of experts of different fields in figuring out what such wellbeing consists of, in the case of everyone. For the proponent of the communal tradition, this is where utilitarianism steps too far: who are these people to tell what wellbeing is for *us*?

**Figure 1.**
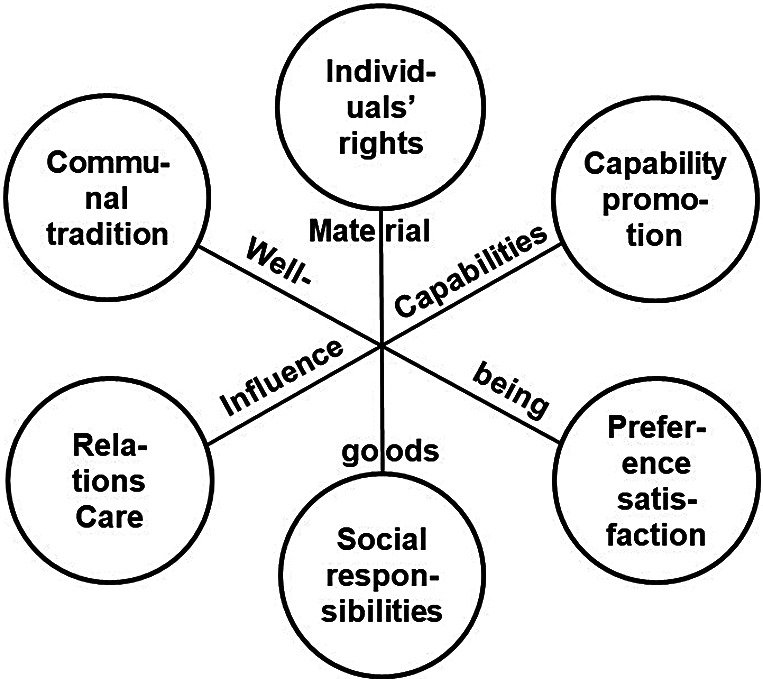
Theories of justice and axes of the good.

In this way, the map Häyry provides can be enriched with a new set of axes linking opposing theories of justice in terms of their fundamental conception of the good (Figure 1). For Häyry’s liberal utilitarianism, this might bring both bad and good news. The bad news is that the connection between the good and justice in the utilitarian account is not as exceptional as the utilitarian might think—or, better put, all accounts of justice stand on a par, each with an answer to the question of the good (or the “what”). However, it may still be that liberal utilitarianism still fares better than its competitors do *when these views of the good are put into play.* Does it? A reconsideration of large-scale challenges to the good in terms of these additional constructs is in order.

## From Justice to the Good—And Back

Let me engage in a bit of speculation. In our societies, there seems to be a tendency to shift back and forth between an emphasis on the good and justice. In “normal” times, when there is plenty of basic goods in the offing, we concentrate on justice: present demands for redistribution, reconsider mechanisms of burdens and benefits, and so on. However, when the basic goods become scarce or are threatened by sudden changes, we seem to be less interested in justice—say, distribution—and more interested in preserving what we already had. There is no way for me to prove this point. But think of the developments that took place in the aftermath of 9/11 and other terrorist attacks, or the current coronavirus crisis. The demands for security and health—or some other basic goods—occasionally seem to trump all considerations of justice. We demand to be provided with food, or jobs, or safety, or healthcare, even at the expense of rights, liberties, principles of just distribution, and so on. Once the exceptional times pass, we may return to the usual considerations of justice. It may even be that, once some good is under severe threat, we may be prepared to move to another conception of the good. When normal times return—when there again appears to be plenty—we may return to calls for justice that align with an account of justice that is different from the one that was dominant before the crisis.

The COVID-19 pandemic and resulting global crisis is a case in point. In a matter of weeks, our most central concern as individuals, groups and nations has become wellbeing—instead of the distribution of material wealth and economic gain, or the development of capabilities, participation, and influence of everyone or particular groups within our societies. Although the elevated concern for public health continues to be questioned based on its detrimental effects on goods of other kinds—material goods, such as salaries and corporate income, and the development of capabilities and opportunities provided by education, among other things—such voices are exceptionally muted. The scope of effects on wellbeing considered has expanded, however. At first concentrated on the effects of and responses to a single contagious disease, we are now focused on attempts to predict the various unintended effects that the current restrictions result in, mostly in terms of health (including mental health). Governmental institutions for health and well-being and experts in various relevant fields have taken the lead in recommending (and in some cases, deciding on) measures.^9^ ^,^^10^

In this fashion, the coronavirus crisis appears to produce two shifts. First, we have entered a time of crisis where the good, under threat, has eclipsed justice as a primary concern. Second, because of the nature of the current crisis, the good is identified with wellbeing rather than with material or financial good, or capabilities, influence, even human rights. Expert opinion is relied upon in large-scale efforts to maximize wellbeing, adjusting the demands of public health and other aspects of wellbeing with one another.^11^ All of this seems like a clear relocation of our conceptions of the good to a utilitarian direction. Perhaps, after the worst crisis is over, even our conception of justice may shift accordingly. Liberal utilitarianism may be the way forward, as we have finally (or again) realized the timeliness of broad considerations of wellbeing.

However, as just pointed out, wellbeing is a view of the good that may be shared across the map of justice, especially in the corner opposite to utilitarianism. Indeed, the actions and measures taken and results—in terms of health and wellbeing—are already estimated mainly on a national level. This is, of course, largely due to the political mechanisms of governance at hand: governmental institutions are designed to advise particular nations or states, and their recommendations pertaining to policy and action are intended to be implemented at a national level. However, we can already see that the role of international and global organizations was immediately diminished by the time the crisis hit several Western nations. In addition, we can also notice new elements of rivalry between nations in stocking up on essential equipment, and even in considering the devastating tallies of people hospitalized and dead in consequence of the pandemic. In particular, there appears to be little concern expressed about the spread of the disease in developing countries, and the potential devastating consequences of the pandemic in impoverished countries; very few measures have been taken against such developments by richer nations. Even if wellbeing has now become the foremost good, it is often considered from a positionalist and communitarian perspective, as opposed to the universal ambitions of liberal utilitarianism.

For contrast, think of the impending climate catastrophe. Before the current pandemic, we were not ready to make sacrifices in favor of major changes: economical and material considerations, as well as considerations of human agency and capabilities, largely trumped the concerns for future wellbeing that climate change brought about. The current shift toward a conception of good more closely aligned with utilitarian ideas might offer a glimmer of hope for a reconsideration of our priorities also with respect to the changing climate. Certainly, many drastic measures just recently considered impossible have become everyday policy, as the threat is perceived as immediate and severe enough.

However, as we have seen, this kind of conception of the good may unite utilitarianism and more parochial views on the left side of Häyry’s map. Instead of a universal demand for climate good, in other corners, consequences on wellbeing may be considered only extremely locally. A changing climate appears to have no winners, but many stand to lose more than others. Many political movements have already proclaimed that climate change—although real and global—is not really among “our” concerns, perhaps because of the small role that “our” limited community can play in curbing it, or because its effects on local wellbeing appear relatively limited, or both. Even if the COVID-19 pandemic pushes us to renewed appreciation of wellbeing—and to a reconsideration of climate action for the sake of wellbeing—we may do so equipped not with a utilitarian account of justice, but a competing, parochial one.

## Conclusion

In defending his liberal utilitarian view of justice, Häyry puts some weight on the perceived shortcomings of competing accounts in considering long-term future good. I have argued that liberal utilitarianism enjoys no such immediate benefit when put to play as an account of justice: it is no more immediately connected to addressing long-term issues of the good than (at least reconstructed versions of) competing accounts. Although utilitarianism is concerned with future good—I have suggested—all the theories of justice may at least provisionally be connected with different accounts of the good, enriching the map of competing accounts that Häyry has provided. Moreover, and perhaps to a surprising extent, accounts of justice in “opposite” corners may be united in their broad conception of the good, or of *what* is to be justly distributed, although they clearly differ in their views of to whom (and on what basis) such benefits and burdens belong. The current COVID-19 pandemic and resulting crisis looks to have at least momentarily elevated wellbeing to a position of primary value, at the expense material or financial good, capabilities, influence, and even human rights. This development may remain in place even as the current crisis is over; it might also give some reason for optimism about increasing climate action. However, quite opposite to the broad universal ambitions of liberal utilitarianism, such future concern for wellbeing may also take an increasingly parochial guise.

## Notes

1.

Rawls J . A Theory of Justice. Revised edition. Harvard: Belknap Press; 1979, 1991, at chap. 26.

2.

Häyry M . Just better utilitarianism. Cambridge Quarterly of Healthcare Ethics 2021;30(2). 10.1017/S0963180120000882.33283691

3.

Häyry M . Liberal Utilitarianism and Applied Ethics. London: Routledge; 1994.

4. See note 2, Häyry 2021.

5. For discussion, see Bognar G . Can the maximin principle serve as a basis for climate change policy? The Monist 2011;94:329–48.

6.

Mill JS . Utilitarianism. 7th ed. London: Longmans, Green and Co.; 1879, at chap. 5. Available online: http://www.gutenberg.org/files/11224/11224-h/11224-h.htm; Sidgwick H. *The Methods of Ethics.* 7th ed (original 1907). London: Macmillan; 1922, at chaps. 5 and 6. Available online at https://www.gutenberg.org/files/46743/46743-h/46743-h.htm.

7.

Häyry M . Doctrines and dimensions of justice. Cambridge Quarterly of Healthcare Ethics 2018;27:188–216, at 207–12.2950911910.1017/S096318011700055X

8. See note 7, Häyry 2018.

9.

Rydenfelt H . Democracy and moral inquiry: Problems of the methodological argument. Transactions of the Charles S. Peirce Society 2019;55(2):254–72.

10.

Rydenfelt H . Pragmatism, social inquiry and the method of democracy In: Holma K , Kontinen T , eds. Practices of Citizenship in East Africa. Perspectives from Philosophical Pragmatism. London: Routledge; 2019:29–43. This and Rydenfelt 2019 (see note 9) discuss the relationship between (expert-driven) social inquiry and democracy. Interestingly, it appears that social inquiry is likely worst conducted in times of crises, but such times are to be exactly when our largest social experiments actually take place.

11. Häyry M. The COVID-19 pandemic: A month of bioethics in Finland. *Cambridge Quarterly of Healthcare Ethics* 2021;30(1):114–22. https://doi.org/10.1017/S0963180120000432.

